# Heterologous expression of human cytochrome P450 2S1 in *Escherichia coli* and investigation of its role in metabolism of benzo[*a*]pyrene and ellipticine

**DOI:** 10.1007/s00706-016-1738-2

**Published:** 2016-03-30

**Authors:** Iveta Mrízová, Michaela Moserová, Jan Milichovský, Miroslav Šulc, René Kizek, Kateřina Kubáčková, Volker M. Arlt, Marie Stiborová

**Affiliations:** Department of Biochemistry, Faculty of Science, Charles University, Albertov 2030, 128 40 Prague 2, Czech Republic; Department of Chemistry and Biochemistry, Faculty of Agronomy, Mendel University in Brno, 613 00 Brno, Czech Republic; Department of Oncology, 2nd Faculty of Medicine, Charles University and University Hospital Motol, V Uvalu 84, 150 06 Prague 5, Czech Republic; Analytical and Environmental Sciences Division, MRC-PHE Centre for Environment and Health, King’s College London, London, SE1 9NH UK

**Keywords:** Enzymes, Coenzymes, High pressure liquid chromatography

## Abstract

**Abstract:**

Cytochrome P450 (CYP) 2S1 is “orphan” CYP that is overexpressed in several epithelial tissues and many human tumors. The pure enzyme is required for better understanding of its biological functions. Therefore, human CYP2S1 was considered to be prepared by the gene manipulations and heterologous expression in *Escherichia coli*. Here, the conditions suitable for efficient expression of human CYP2S1 protein from plasmid pCW containing the human CYP2S1 gene were optimized and the enzyme purified to homogeneity. The identity of CYP2S1 as the product of heterologous expression was confirmed by dodecyl sulfate–polyacrylamide gel electrophoresis, Western blotting, and mass spectrometry. To confirm the presence of the enzymatically active CYP2S1, the CO spectrum of purified CYP2S1 was recorded. Since CYP2S1 was shown to catalyze oxidation of compounds having polycyclic aromatic structures, the prepared enzyme has been tested to metabolize the compounds having this structural character; namely, the human carcinogen benzo[*a*]pyrene (BaP), its 7,8-dihydrodiol derivative, and an anticancer drug ellipticine. Reaction mixtures contained besides the test compounds the CYP2S1 enzyme reconstituted with NADPH:CYP reductase (POR) in liposomes, and/or this CYP in the presence of cumene hydroperoxide or hydrogen peroxide. High performance liquid chromatography was employed for separation of BaP, BaP-7,8-dihydrodiol, and ellipticine metabolites. The results found in this study demonstrate that CYP2S1 in the presence of cumene hydroperoxide or hydrogen peroxide catalyzes oxidation of two of the test xenobiotics, a metabolite of BaP, BaP-7,8-dihydrodiol, and ellipticine. Whereas BaP-7,8,9,10-tetrahydrotetrol was formed as a product of BaP-7,8-dihydrodiol oxidation, ellipticine was oxidized to 12-hydroxyellipticine, 13-hydroxyellipticine, and the ellipticine *N*^2^-oxide.

**Graphical abstract:**

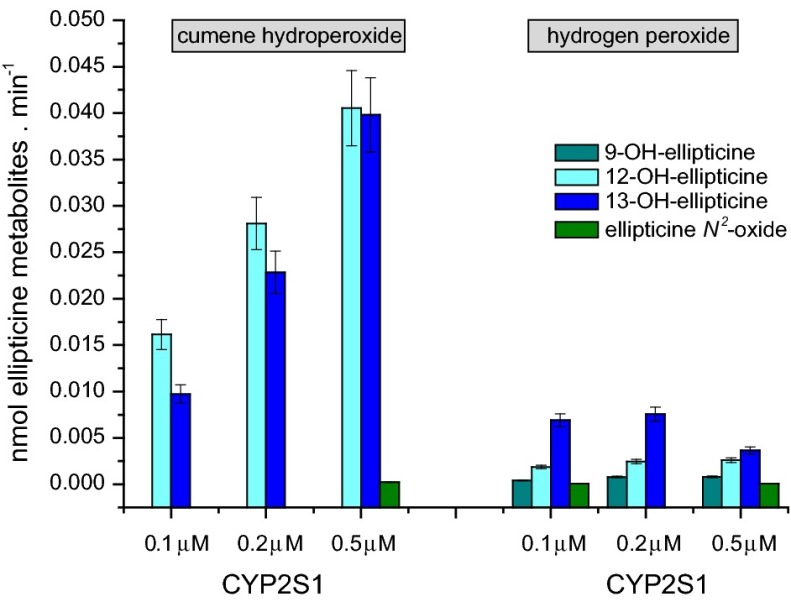

**Electronic supplementary material:**

The online version of this article (doi:10.1007/s00706-016-1738-2) contains supplementary material, which is available to authorized users.

## Introduction

Cytochrome P450 (CYP, EC 1.14.14.1) is a superfamily of hemoproteins distributed widely throughout nature, involved in metabolism of a broad variety of substrates and catalyzing a variety of interesting chemical reactions [1–3]. The CYP enzymes are a component of a mixed function oxidase (MFO) system located in the membrane of the endoplasmic reticulum that beside the CYPs also contains other enzymes such as NADPH:CYP oxidoreductase (POR), and cytochrome *b*_*5*_ accompanied by its NADH:cytochrome *b*_*5*_ reductase [1–5].

A variety of CYP enzymes (e.g., CYP5, 8, 11, 17, 19, 21, 24, 26, and 27) are used by mammals for the synthesis of important endogenous compounds such as steroids and eicosanoids, besides their function in the catabolism of natural products [1, 2]. Of the remainder of the mammalian CYPs, a relatively small set of these enzymes accounts for most of metabolism of drugs (i.e., human CYP1A2, 2C9, 2C19, 2D6, and 3A4). Another group of human CYPs (i.e., human CYP1A1, 1A2, 1B1, 2A6, 2E1, and 3A4) is involved in the metabolism of most pro-toxicants and pro-carcinogens that are CYP substrates [1, 3, 6, 7]. All these data indicate that functions of most CYP enzymes are well known. However, of 57 human CYP enzymes, 13 remain classified as “orphans”, because their functions are largely unknown [8–11]. Among them, CYP2S1 was found to be induced by the aryl hydrocarbon receptor (AHR) ligands, by hypoxia via hypoxia-inducible factor 1 and by all-*tran*s-retinoic acid [12, 13]. This enzyme is expressed in epithelial cells of tissues exposed to the environment (i.e., skin, respiratory, urinary, and gastrointestinal tracts), and also in many tumors of epithelial origin [10, 12]. Interestingly, CYP2S1 was described that cannot use NADPH for oxidative metabolism of some substrates, because of its inability to accept electrons from the NADPH/POR system [11, 14]. However, the recent results of Guengerich with collaborators show that in the case of CYP2S1-mediated metabolism of 1,4-bis[[2-(dimethylamino-*N*-oxide)ethyl]amino]-5,8-dihydroxyanthra-cene-9,10-dione by CYP2S1, POR is capable of reducing this CYP [15].

Recently, we have found that a human carcinogen benzo[*a*]pyrene (BaP) [16] and an anticancer drug ellipticine [17–19] might be, beside classical CYPs that accept electrons transferred from the NADPH/POR system, oxidized also by other CYP enzymes, whose activities are not dependent on POR [17, 18, 20–24]. We suggested that the CYP2S1 enzyme, which was shown to catalyze the oxidation of compounds having polycyclic aromatic structures (such as BaP) also without participation of POR [8, 10, 11, 14], might be one of the such enzymes. Therefore, to confirm this suggestion, the CYP2S1 efficiency to oxidize BaP and ellipticine should be investigated in detail. However, because purification of CYP2S1 from natural biological materials is experimentally very difficult, heterologous expression was recently employed to obtain the biological active CYP2S1 [9–11]. In this study, we describe an improved method for heterologous expression of human CYP2S1 in a prokaryotic expression system of *Escherichia coli* cells as well as an efficient procedure for its purification to homogeneity. The purified enzyme either reconstituted with POR in liposomes or in the presence of cumene hydroperoxide/hydrogen peroxide in vitro was utilized to investigate its catalytic activity to oxidize carcinogenic BaP, its 7,8-dihydrodiol metabolite, and an anticancer drug ellipticine.

## Results and discussion

### Expression of human CYP2S1 in *E. coli* and its purification

When the procedure for heterologous expression of human CYP2S1 construct in *E. coli* DH5α cells described by [9] was used, the CYP2S1 production was only very low (less than 70 nmol CYP per dm^3^). Moreover, even though the co-expression of the molecular chaperon GroEL/ES that is a suitable method to elevate the CYP2S1 expression [9] was utilized, no dramatic increase in its expression was found. Therefore, we examined other modifications of the procedure to improve the CYP2S1 expression. First, the effect of volume of the cultured growth media from 50 to 500 cm^3^ in different size Erlenmeyer flasks was tested. The level of expression CYP2S1 increased up to 200 nmol CYP per dm^3^ when the 100 cm^3^ of cultured media in 500 cm^3^ Erlenmeyer flask was used, compared with 50 or 500 cm^3^ of growth TB media in 250 cm^3^ and/or 2 dm^3^ Erlenmeyer flasks, respectively. Second, in addition to this procedure modification, the cell growth time was found to play a role in the production of CYP2S1, and even in its quality. The maximum levels of expressed CYP occurred after 24-h cultivation, at shaking speed of 190 rpm and 29 °C; higher time of cultivation (up to 40 h) did not result to elevated levels of CYP produced. It, moreover, led to changes in a CYP structure, forming its degraded form, cytochrome P420. Therefore, the 24-h cell cultivation was used in this study and the cells prepared by this procedure were utilized for CYP2S1 purification.

**Fig. 1:**
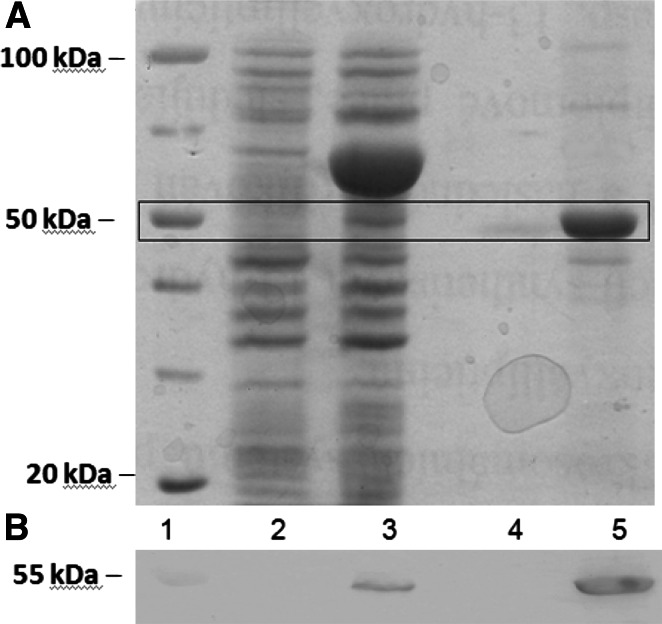
SDS-PAGE (**a**) and Western blotting (**b**) of heterologous expression products in *E. coli* and fractions obtained during purification of CYP2S1*. Lane 1* protein *M*_*r*_ marker, *2* sample before induction by IPTG, *3* production of proteins after 24 h, *4* Ni^2+^-nitriloacetic acid agarose purified fraction, *5* a final concentrated protein sample

Solubilization of *E. coli* membranes containing CYP2S1 was achieved by 1 % 3-[(3-cholamidopropyl)dimethylammonio]-1-propanesulfonate hydrate (CHAPS) (w/v) present in the solubilization buffer. The resulting supernatant was loaded onto a column of Ni^2+^-nitriloacetic acid agarose (Ni–NTA agarose) and CYP2S1 was eluted with potassium phosphate buffer containing 300 mmol dm^−3^ imidazole. Using the sodium dodecyl sulfate–polyacrylamide gel electrophoresis (SDS-PAGE), the purified, detergent-free CYP2S1 (see “Experimental”), was shown to be electrophoretically homogeneous, having a molecular mass of ~50 ± 5 kDa (Fig. 1a). The CYP2S1 identity was proved by Western blotting, using the chicken polyclonal antibodies against CYP2S1 [25] (Fig. 1b) and by mass spectrometry (Supplementary Table 1 and Supplementary Scheme 1). The specific content of CYP2S1 was estimated to be 4.5 nmol per mg protein, based on a bicinchoninic acid colorimetric protein estimation method.

### Spectral properties of prepared CYP2S1

**Fig. 2:**
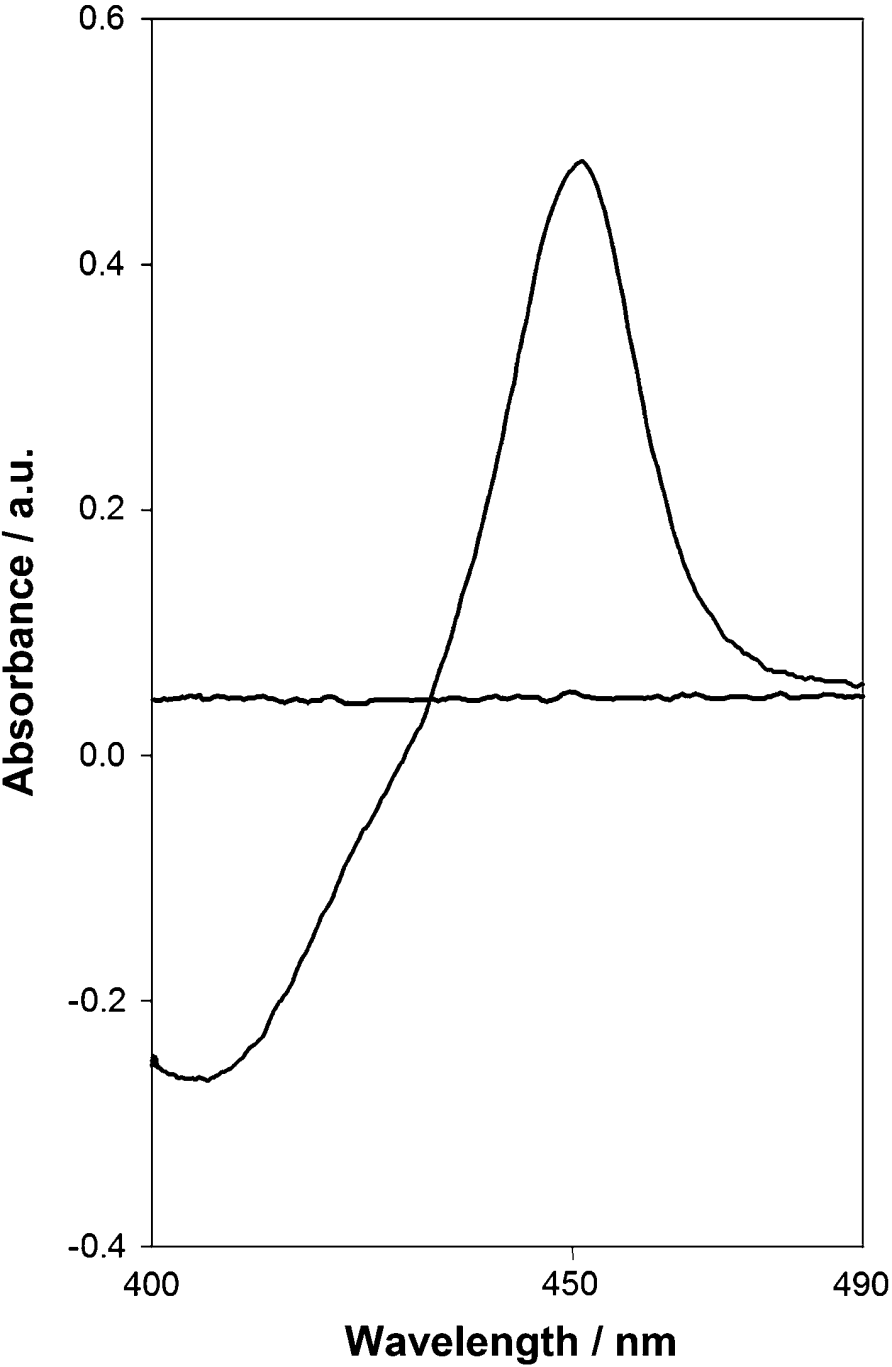
The CO-spectrum of purified human CYP2S1. Fe^2+^–CO vs. Fe^2+^ difference spectrum

The carbon monoxide (CO)-spectrum (see “Experimental”) of prepared human CYP2S1 was recorded (Fig. 2). This spectrum was free of cytochrome P420, indicating the correct fold and the high quality of the prepared CYP2S1 enzyme.

### Enzyme activity—oxidation of BaP, BaP-7,8-dihydrodiol, and ellipticine by prepared CYP2S1

To evaluate enzymatic activity of purified CYP2S1, two systems were utilized: (1) CYP2S1 reconstituted with POR in liposomes and (2) CYP2S1 in the presence of cumene hydroperoxide and/or hydrogen peroxide as cofactors. Since BaP and its 7,8-dihydrodiol derivative were found as substrates of CYP2S1 [10, 11], they were used as model substrates of these CYP2S1 systems. In addition, because a suggested participation of CYP2S1 in metabolism of an anticancer drug ellipticine [17, 23, 24] has not been examined as yet, its potency to oxidize this drug was investigated, too. The enzyme activity of CYP2S1 to metabolize these compounds in both systems was compared with that of the CYP1A1 and/or 1B1 enzymes, for which the test compounds are excellent substrates [3, 21, 22, 26–28].

**Fig. 3:**
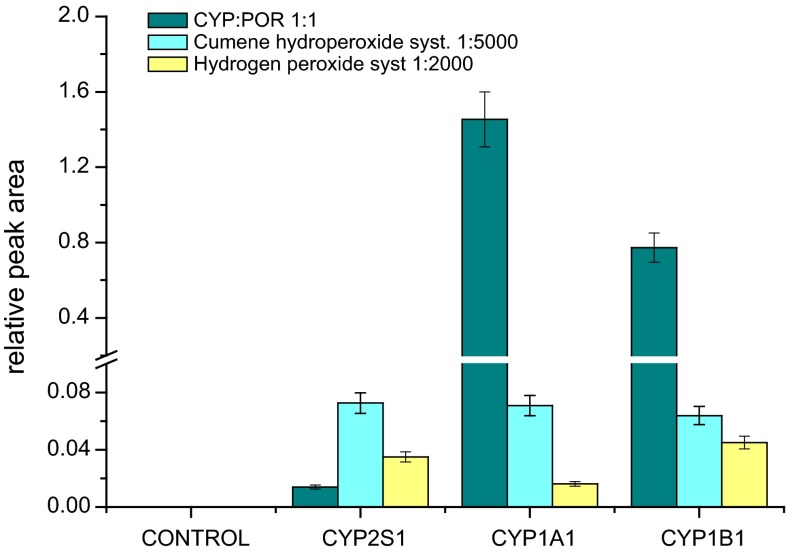
Formation of BaP-7,8,9,10-tetrahydrotetrol by human CYP2S1 in different systems. Human CYP1A1 or CYP1B1 known to oxidize BaP- 7,8-dihydrodiol to BaP-7,8,9,10-tetrahydrotetrol were used as positive controls. Data shown are averages and standard deviation from three parallel measurements

Utilizing BaP as a compound that was described by Bui and Hankinson [10] to be a substrate of CYP2S1, no metabolites were detectable in both CYP2S1 systems under the conditions used in our experiments. This finding, namely, the result that BaP is not a substrate of CYP2S1, is in line with the results of Guengerich and collaborators [9], whose detected almost no CYP2S1 activity to oxidize BaP, too. In contrast, HPLC used to analyze metabolism of one of the BaP metabolites, BaP-7,8-dihydrodiol, with either CYP2S1 systems showed their effectiveness to oxidize this xenobiotic (see Supplementary Fig. 1 for CYP2S1 with cumene hydroperoxide); BaP-7,8,9,10-tetrahydrotetrol was identified as the reaction product (Supplementary Fig. 2). As shown in Fig. 3, CYP1A1 or 1B1 reconstituted with POR in liposomes oxidized BaP-7,8-dihydrodiol with up to more than two orders of magnitude higher effectiveness than CYP2S1 reconstituted with POR. On the contrary, CYP2S1 in the presence of cumene hydroperoxide or hydrogen peroxide generated up sevenfold higher levels of BaP-7,8,9,10-tetrahydrotetrol than CYP2S1 reconstituted with POR. In these systems, the CYP2S1 enzyme exhibited essentially the same (and/or even higher) effectiveness in this reaction than analogous systems of human CYP1A1 or 1B1 (Fig. 3). Essentially no BaP-7,8-dihydrodiol metabolite (BaP-7,8,9,10-tetrahydrotetrol) was detectable when the NADPH-generating system or cumene hydroperoxide/hydrogen peroxide cofactors were omitted from the incubation mixtures (data not shown). The results found indicate that prepared human CYP2S1 is enzymatically active enzyme, being more effective in BaP-7,8-dihydrodiol oxidation in the presence of cumene hydroperoxide or hydrogen peroxide than in the system where CYP2S1 is reconstituted with POR. They also demonstrate that by its potency to oxidize BaP-7,8-dihydrodiol, this CYP can increase the overall metabolism of BaP catalyzed by other CYPs, including CYP1A1 and 1B1, the most important CYPs oxidizing this carcinogen [20, 29].

**Fig. 4:**
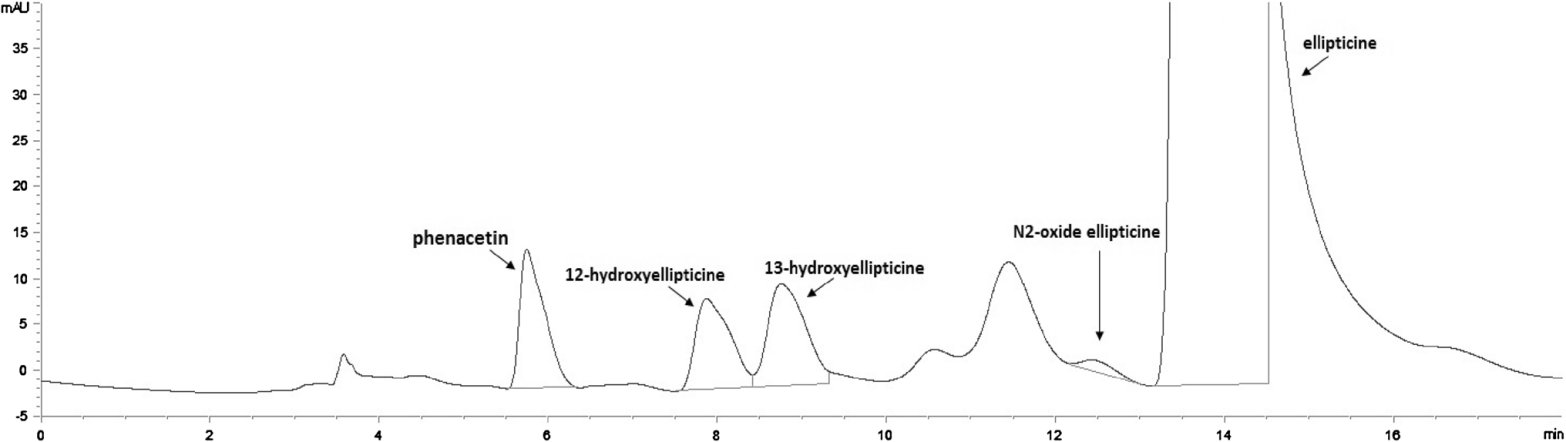
HPLC analysis of ellipticine metabolites formed by human recombinant CYP2S1 in the presence of cumene hydroperoxide

**Fig. 5:**
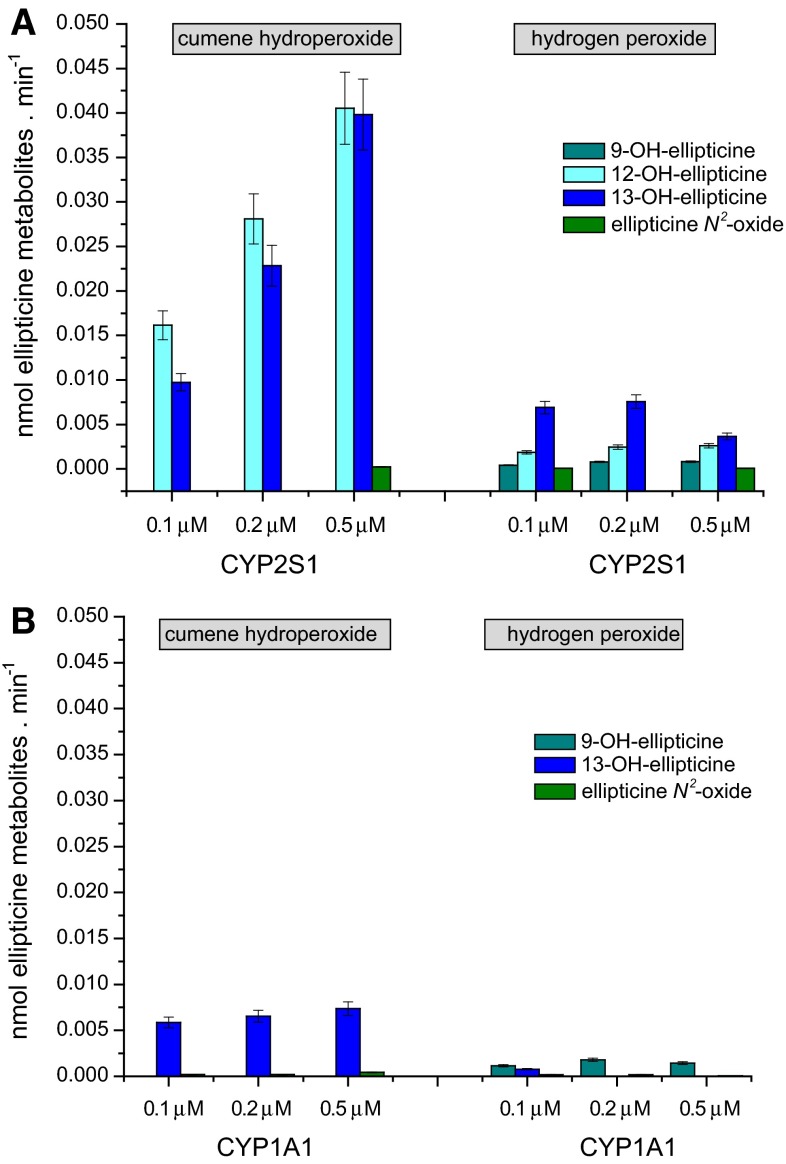
Oxidation of ellipticine by human recombinant CYP2S1 (**a**) and CYP1A1 (**b**) expressed in *E. coli* with cumene hydroperoxide or H_2_O_2_. Data shown are averages and standard deviation from three parallel measurements

In the case of ellipticine, only the systems of CYP2S1 containing cumene hydroperoxide and/or hydrogen peroxide were capable of oxidizing this drug (Figs. 4, 5), whereas this CYP reconstituted with POR in liposomes in the presence of NADPH-generating system was without such activity (data not shown). Cumene hydroperoxide was an up to one order of magnitude more effective cofactor for ellipticine oxidation than hydrogen peroxide (Fig. 5a). Of the ellipticine metabolites, 12-hydroxyellipticine and 13-hydroxyellipticine were the major metabolites formed by this CYP2S1 system, while 9-hydroxyellipticine and the ellipticine *N*^2^-oxide were the minor ones (Fig. 5a). Negligible amounts of these ellipticine metabolites were generated by CYP2S1 without cofactors (data not shown). The formation of 12-hydroxyellipticine and 13-hydroxyellipticine is the pharmacologically important feature, because these metabolites generate two major deoxyguanosine adducts in DNA [17–19, 30, 31, 32] that are responsible for ellipticine anticancer activity [17–19, 33, 34]. The levels of activation metabolites were increased with increasing concentrations of CYP2S1 with cumene hydroperoxide, while essentially no such effects were found when hydrogen peroxide was used as a cofactor (Fig. 5a). The only low amounts of the ellipticine metabolites were generated by the system of human CYP1A1 in the presence of both peroxides, 12-hydroxyellipticine was even not produced by this CYP at all (Fig. 5b). The system of human CYP1A1 reconstituted with POR in liposomes was not examined in this work, because its efficiency to oxidize this drug was investigated previously in detail; 9-hydroxyellipticine, 7-hydroxyellipticine, and 13-hydroxyellipticine were formed as the major metabolites in this CYP1A1 system, while the ellipticine *N*^2^-oxide as a minor product and 12-hydroxyellipticine was not a product of ellipticine oxidation by this enzyme [19, 31, 32]. A pattern of the metabolites formed by this CYP1A1 system is hence quite different from that generated by this enzyme in the presence of either test peroxides (Fig. 5b). The results found demonstrate the effectiveness of CYP2S1 in ellipticine oxidation and confirmed the suggestion of its participation in ellipticine metabolism catalyzed by CYP without POR [17, 23, 24]. Because of the high expression of CYP2S1 in many tumor cells, the results also emphasized its role in metabolism of ellipticine in cancer tissues that was found previously [17, 19, 33, 34].

## Conclusion

Since functions of many “orphan” CYPs are largely unknown [8–11], their investigation is a challenge for research of many laboratories. Therefore, in this study we examined a function of “orphan” CYP2S1, the enzyme which is able to metabolize several substrates via the NADPH/POR-independent activity [9–11]. For such a study, we prepared enzymatically active human recombinant CYP2S1 and further utilized to evaluate its potency to oxidize one of the anticancer drugs, ellipticine. This antitumor agent was chosen, because it was found that it can be metabolized also by the CYP enzymes that do not need the NADPH/POR system, and we suggested that CYP2S1 might be one of these CYPs [17, 23, 24]. Our data demonstrate that CYP2S1 is indeed capable of oxidizing ellipticine, in the systems containing the peroxide cofactors, therefore, via the NADPH/POR-independent activity. CYP2S1 in the presence of cumene hydroperoxide oxidizes ellipticine predominantly to its activation metabolites, 12-hydroxyellipticine and 13-hydroxyellipticine, the metabolites responsible for its pharmacological efficiencies that result from formation of covalent ellipticine-derived DNA adducts. These adducts were found to be generated in rat lung and kidney in vivo, indeed even without participation of the NADPH/POR system [17, 18, 23, 24].

Because CYP2S1 can be expressed in several tumor cells sensitive to ellipticine [10, 25, 32–36], the data found in this work suggest that this CYP can contribute to the ellipticine antitumor activity in these cells. This suggestion need, however, to be explored by further studies. Utilization of the tumor cells highly expressing CYP2S1 [10, 25] is planned to be utilized for such studies.

## Experimental

Ellipticine and BaP were from Sigma Chemical Co (St Louis, MO, USA). These and other chemicals used in the experiments were of analytical purity or better. (±)-*Trans*-7,8-dihydroxy-7,8-dihydrobenzo[*a*]pyrene (BaP-7,8-dihydrodiol) was prepared at the Biochemical Institute for Environmental Carcinogenesis, Germany, as described [37].

*Escherichia coli* DH5α cells with the *CYP2S1* gene were a gift of Professor F.P. Guengerich (Vanderbilt University School of Medicine, Nashville, Tennessee, USA). It should be noted that optimization of the conditions suitable for production of plasmid pCW containing the CYP2S1 gene, was carried out in his laboratory and further used for expression of CYP2S1 [9]. It was necessary to modify the C-terminal end of the CYP2S1 cDNA by Histidyl-5-Tag and the N-terminal end before the well-conserved proline-rich region (see [9] for detail).

Human CYP1A1, 1B1 and POR were prepared by heterologous expression in *E. coli* in our laboratory (Department of Biochemistry, Faculty of Sciences, Charles University, Prague, Czech Republic), by J. Milichovský, essentially in the same manner as described previously [38–40].

### Expression of human CYP2S1 in *E. coli*

*Escherichia coli* cultivation was carried out essentially as described previously [9, 41–43]. A single bacterial colony was picked, and the bacteria were grown overnight in 15 cm^3^ of a Luria Broth (LB) liquid medium (Sigma Chemical Co, St Louis, MO, USA) containing 100 mg cm^−3^ ampicillin, and 50 mg cm^−3^ kanamycin at 37 °C with shaking at 200 rpm. The overnight culture was inoculated 1:100 into 100 cm^3^ of a Terrific Broth (TB) medium (Sigma Chemical Co, St Louis, MO, USA) containing 100 mg cm^−3^ ampicilin, 50 mg cm^−3^ kanamycin, and 0.025 % (v/v) of a mixture of trace elements (27 g FeCl_3_·6H_2_O, 2.0 g ZnCl_2_·4H_2_O, 2.0 g CaCl_2_·6H_2_O, 2.0 g Na_2_MoO_4_, 1.0 g CaCl_2_·2H_2_O, 1.0 g CuCl_2_, 0.5 g H_3_BO_3_, and 100 cm^3^ concentrated HCl per 1 dm^3^ of distilled water) in a 500 cm^3^ flask. Sixteen flasks of cultures were incubated for ~4 h at 37 °C with shaking at 220 rpm until they attained an OD_600_ of 0.5–0.7, then were supplemented with 1 mmol dm^−3^ isopropyl *β*-D-1-thiogalactopyranoside (IPTG) medium (Sigma Chemical Co, St Louis, MO, USA), 1 mg cm^−3^ arabinose, and 0.5 mmol dm^−3^ a heme precursor, *δ*-aminolevulinic acid (*δ*-ALA) (Sigma Chemical Co, St Louis, MO, USA), and cultured for 24 h at 29 °C with shaking at 190 rpm. Induction of GroEL/ES and CYP2S1 synthesis was achieved in the presence of arabinose and IPTG, respectively. The expression level of CYP2S1 was monitored at 12 and 24 h.

*Escherichia coli* membranes were prepared as described previously [44]. All steps were done at 4 °C. The cells were harvested by centrifugation at 5000*g* for 20 min. Pellets were resuspended in 1:20 vol of Tris/sucrose/ethylenediaminetetraacetic acid (EDTA) (TSE) buffer (10 mmol dm^−3^ Tris–acetate, pH 7.4, 500 mmol dm^−3^ sucrose, 0.5 mmol dm^−3^ EDTA). Lysozyme was added to a concentration of 0.2 mg cm^−3^, and the suspensions were diluted twofold with distilled H_2_O before incubation on ice for 30 min. The resulting spheroplasts were sedimented at 4000 g at 4 °C for 20 min, and resuspended in 100 mmol dm^−3^ potassium phosphate buffer, pH 7.4, containing 6 mmol dm^−3^ magnesium acetate, 20 % glycerol (v/v), and 10 mmol dm^−3^*β*-mercaptoethanol (ME). The protease inhibitors, phenylmethylsulfonyl fluoride (PMSF), aprotinin, leupeptin, and bestatin (all Sigma Chemical Co, St Louis, MO, USA) were added to a final concentration of 1 mmol dm^−3^, 1 µg cm^−3^, 2, and 1 μmol dm^−3^, respectively. Suspensions of spheroplasts were sonicated ten times for 30 s each, on ice, and centrifuged at 10,000*g* at 4 °C for 20 min. Supernatants were centrifuged at 108,000*g* at 4 °C for 65 min. Sedimented membrane fractions were resuspended in 100 mmol dm^−3^ potassium phosphate buffer, pH 7.4, containing 6 mmol dm^−3^ magnesium acetate, 20 % glycerol (v/v), and 10 mmol dm^−3^ ME. The membrane preparation was stored at −70 °C until use. The reddish pellets represent the CYP containing membrane fraction.

### Purification of recombinant human CYP2S1

Membrane fractions of *E. coli* were diluted up to 2 mg cm^−3^ of protein concentration with 100 mmol dm^−3^ potassium phosphate buffer, pH 7.4, containing 20 % glycerol (v/v), 0.5 mol dm^−3^ NaCl, 10 mmol dm^−3^ ME (solubilization buffer) and solubilized with 1.0 % CHAPS (Sigma Chemical Co, St Louis, MO, USA) (w/v). The detergent was dissolved in solubilization buffer. After stirring for 3 h at 4 °C, the resulting solutions were centrifuged at 108,000*g* at 4 °C for 65 min to eliminate insoluble materials. The supernatants were then applied on a column of Ni–NTA agarose (QIAGEN) (Hilden, Germany) equilibrated with the solubilization buffer [9]. After washing of the column with 10 volumes of 100 mmol dm^−3^ potassium phosphate buffer, pH 7.4, containing 20 % glycerol (v/v), 0.5 mol dm^−3^ NaCl, 0.5 % CHAPS (w/v) and 10 mmol dm^−3^ imidazole, the CYP2S1 enzyme was eluted with 100 mmol dm^−3^ potassium phosphate buffer, pH 7.4, containing 20 % glycerol (v/v), 0.5 mol dm^−3^ NaCl, 0.5 % CHAPS (w/v) and 300 mmol dm^−3^ imidazole. The CHAPS was removed by extensive dialysis against 200-fold volume of 100 mmol dm^−3^ potassium phosphate buffer, pH 7.4 containing 20 % glycerol (v/v), 0.1 mmol dm^−3^ EDTA, and 0.1 mmol dm^−3^ dithiothreitol (DTT) (dialysis buffer). In the last step of dialysis, the dialysis buffer without DTT was used. The final fraction of CYP2S1 was concentrated by ultrafiltration using an YM-30 membrane (Millipore). SDS-PAGE was used to assess the final protein purity.

### Determination of CYP and protein contents

As found by Omura and Sato [45], when CYP is in the reduced state and complexed with CO, it exhibits the classic CO difference spectrum with a maximum at 450 nm. This spectral property is employed for the specific estimation of CYP content and for evaluation whether the purified enzyme exists in a correctly folded protein state. Therefore, the concentration of CYP was estimated by this method, described by Omura and Sato [45] that is based on the absorption of the complex of reduced CYP with CO. The CYP2S1 content was calculated using an extinction coefficient of 91 [mmol dm^−3^]^−1^ cm^−1^. Protein concentrations were estimated using a bicinchonic acid assay (BCA, ThermoFisher Scientific, USA) with bovine serum albumin as a standard [46].

### Determination of CYP2S1 protein levels by Western blotting

Samples containing subcellular fractions from bacteria or the purified protein were subjected to electrophoresis on the 10 % polyacrylamide gel and transferred onto a polyvinylidene fluoride (PVDF) membrane as reported [22, 47]. The membranes were then exposed overnight at 4 °C to the chicken polyclonal antibodies against CYP2S1 prepared as described previously [25] and the antigen–antibody complex was visualized with an alkaline phosphatase-conjugated goat anti-rabbit IgG antibody (1:1428, Sigma-Aldrich, USA) and 5-bromo-4-chloro-3-indolylphosphate/nitrobluetetrazolium as chromogenic substrate [48].

### Measurement of CYP2S1 enzyme activities

Enzymatic activity of purified CYP2S1 was analyzed using two systems: (1) CYP2S1 reconstituted with POR in liposomes and (2) CYP2S1 in the presence of cumene hydroperoxide and/or hydrogen peroxide.

Incubation mixtures containing purified CYP reconstituted with human POR contained CYP2S1 (or CYP1A1 or 1B1) and POR. Briefly, CYP was reconstituted as follows (200 pmol CYP with 200 pmol POR, 0.1 mmol cm^−3^ liposomes [dilauroyl phosphatidylcholine, dioleyl phosphatidylcholine, dilauroyl phosphatidylserine (1:1:1) (Sigma Chemical Co, St Louis, MO, USA)], 3 mmol dm^−3^ reduced glutathione, and 50 mmol dm^−3^ HEPES/KOH buffer, pH 7.4) [49]. An aliquot of this mixture was then added to incubation mixtures.

Incubation mixtures used to study metabolism of BaP, BaP-7,8-dihydrodiol, or ellipticine contained 100 mmol dm^−3^ sodium phosphate buffer (pH 7.4), NADPH-generating system (1 mmol dm^−3^ NADP^+^, 10 mmol dm^−3^d-glucose-6-phosphate, 1 U per cm^−3^d-glucose-6-phosphate dehydrogenase, Sigma Chemical Co, St Louis, MO, USA), 0.05 cm^3^ CYP reconstituted system, 10 μmol dm^−3^ BaP or 133 μmol dm^−3^ BaP-7,8-dihydrodiol or 10 μmol dm^−3^ ellipticine (dissolved in 0.005 cm^3^ DMSO) in a final volume of 0.5 cm^3^. The reaction was initiated by adding 0.05 cm^3^ of the NADPH-generating system. Control incubations were carried out either without CYP enzyme or without the NADPH-generating system or without the substrate. After incubation in open tubes (37 °C, 20 min), 0.005 cm^3^ of 1 mmol dm^−3^ phenacetin in methanol was added as an internal standard. The reactions catalyzed oxidation of BaP or BaP-7,8-dihydrodiol and ellipticine by human CYP1A1 was linear up to 60 and 30 min, respectively [26, 28, 49]. Metabolites were extracted twice with ethyl acetate (2 × 1 cm^3^) and evaporated to dryness. The samples were dissolved in 0.025 cm^3^ methanol and metabolites of the tested substrates formed in these systems separated by HPLC. Metabolites of BaP and BaP-7,8-dihydrodiol were separated by HPLC and identities of their structures were carried out as described [28, 49, 50]. HPLC analysis of ellipticine metabolites and identities of their structures were performed as described previously [26, 31, 32].

Incubation mixtures used to study metabolism of BaP, BaP-7,8-dihydrodiol, or ellipticine in the presence of cumene hydroperoxide or hydrogen peroxide contained 100 mmol dm^−3^ sodium phosphate buffer (pH 7.4), 0.1–0.5 μmol dm^−3^ CYP, 10 μmol dm^−3^ BaP or 133 μmol dm^−3^ BaP-7,8-dihydrodiol or 10 μmol dm^−3^ ellipticine (dissolved in 0.005 cm^3^ DMSO) in a final volume of 500 cm^3^. The reaction was initiated by adding cumene hydroperoxide or hydrogen peroxide to their final concentrations of 1 mmol dm^−3^. Control incubations were carried out either without CYP or without peroxides or without substrates. After incubation in open tubes (37 °C, 10 min), 0.005 cm^3^ of 1 mmol dm^−3^ phenacetin in methanol was added as an internal standard. Metabolites were extracted twice with ethyl acetate (2 × 1 cm^3^) and evaporated to dryness. The samples were dissolved in 0.025 cm^3^ methanol and different metabolites formed in these systems were separated by HPLC. Metabolites of BaP, BaP-7,8-dihydrodiol and ellipticine were separated by HPLC and their structure indentified as described above. The peak areas of BaP, BaP-7,8-dihydrodiol, and ellipticine were calculated relative to the peak area of the internal standard (phenacetin), and expressed as relative peak areas.

### Mass spectrometry of purified CYP2S1

The identity and integrity of recombinant protein of human CYP2S1 was verified by MALDI-TOF/TOF (Ultra-FLEX III mass spectrometer, Bruker-Daltonics, Bremen, Germany) mass spectrometer using *α*-cyano-4-hydroxycinnamic acid as a matrix. The protein band from SDS-PAGE was destained, cysteine residues were modified by iodoacetamide, and protein was digested by trypsin endoprotease (Promega Corp., Madison, USA). The resulting peptide mixture was extracted and the MS and MS/MS spectra of corresponding *m*/*z* signals for peptide identity verification were acquired and manually interpreted (54 % of protein sequence coverage) [51].

## Electronic supplementary material

Below is the link to the electronic supplementary material.

Supplementary material 1 (DOCX 132 kb)

## References

[1] Guengerich FP (2001). Chem Res Toxicol.

[2] Guengerich FP (2008). Chem Res Toxicol.

[3] Arlt VM, Henderson CJ, Wolf CR, Stiborova M, Phillips DH (2015). Toxicol Res.

[4] Riddick DS, Ding X, Wolf CR, Porter TD, Pandey AV, Zhang QY, Gu J, Finn RD, Ronseaux S, McLaughlin LA, Henderson CJ, Zou L, Fluck CE (2013). Drug Metab Disp.

[5] Porter TD (2002). J Biochem Mol Toxicol.

[6] Rendic S, Di Carlo FJ (2007). Drug Metab Rev.

[7] Wienkers LC, Heath LC (2005). Nat Rev Drug Dis.

[8] Guengerich FP, Wu ZL, Bartleson CJ (2005). Biochem Biophys Res Commun.

[9] Wu ZL, Sohl CD, Shimada T, Guengerich FP (2006). Mol Pharmacol.

[10] Bui PH, Hankinson O (2009). Mol Pharmacol.

[11] Bui PH, Hsu EL, Hankinson O (2009). Mol Pharmacol.

[12] Smith G, Wolf CR, Deeni YY, Dawe RS, Evans AT, Comrie MM, Ferguson J, Ibbotson SH (2003). Lancet.

[13] Rivera SP, Wang F, Saarikoski ST, Taylor RT, Chapman B, Zhang R, Hankinson (2007) J Biol Chem 282:10881 10.1074/jbc.M609617200 17277313

[14] Bui P, Imaizumi S, Beedanagari SR, Reddy ST, Hankinson O (2011). Drug Metab Dispos.

[15] Xiao Y, Shinkyo R, Guengerich FP (2011). Drug Metab Dispos.

[16] International Agency for Research on Cancer (IARC) (2010). Some non-heterocyclic polycyclic aromatic hydrocarbons and some related exposures. In: IARC Monogr Eval Carcinog Risks Hum 92:1 PMC4781319 21141735

[17] Stiborová M, Rupertová M, Frei E (2011). Biochim Biophys Acta.

[18] Stiborová M, Černá V, Moserová M, Mrízová I, Arlt VM, Frei E (2015). Int J Mol Sci.

[19] Stiborova M, Frei E (2014). Curr Med Chem.

[20] Stiborova M, Moserova M, Cerna V, Indra R, Dracinsky M, Šulc M, Henderson CJ, Wolf CR, Schmeiser HH, Phillips DH, Frei E, Arlt VM (2014). Toxicology.

[21] Arlt VM, Stiborova M, Henderson CJ, Thiemann M, Frei E, Aimova D, Singhs R, da Costa GG, Schmitz OJ, Farmer PB, Wolf CR, Philips DH (2008). Carcinogenesis.

[22] Arlt VM, Poirier MC, Sykes SE, Kaarthik J, Moserova M, Stiborova M, Wolf R, Henderson CJ, Phillips DH (2012). Toxicol Lett.

[23] Stiborová M, Arlt VM, Henderson CJ, Wolf CR, Kotrbová V, Moserová M, Hudecek J, Phillips DH, Frei E (2008). Toxicol Appl Pharmacol.

[24] Stiborova M, Moserova M, Mrazova B, Kotrbova V, Frei E (2010). Neuro Endocrinol Lett.

[25] Hodek P, Hrdinova J, Macova I, Soucek P, Mrizova I, Burdova K, Kizek R, Hudecek J, Stiborova M (2015). Neuro Endocrinol Lett.

[26] Kotrbova V, Mrazova B, Moserova M, Martinek V, Hodek P, Hudecek J, Frei E, Stiborova M (2011). Biochem Pharmacol.

[27] Stiborova M, Indra R, Moserova M, Cerna V, Rupertova M, Martinek V, Eckschlager T, Kizek R, Frei E (2012). Chem Res Toxicol.

[28] Stiborova M, Moserova M, Cerna V, Indra R, Dracinsky M, Šulc M, Henderson CJ, Wolf CR, Schmeiser HH, Phillips DH, Frei E, Arlt VM (2014). Toxicology.

[29] Baird WM, Hooven LA, Mahadevan B (2005). Environ Mol Mutagen.

[30] Stiborová M, Sejbal J, Borek-Dohalská L, Aimová D, Poljaková J, Forsterová K, Rupertová M, Wiesner J, Hudecek J, Wiessler M, Frei E (2004). Cancer Res.

[31] Stiborová M, Poljaková J, Martínková E, Ulrichová J, Simánek V, Dvořák Z, Frei E (2012). Toxicology.

[32] Poljaková J, Frei E, Gomez JE, Aimová D, Eckschlager T, Hrabeta J, Stiborová M (2007). Cancer Lett.

[33] Martinkova E, Dontenwill M, Frei E, Stiborova M (2009). Neuro Endocrinol Lett.

[34] Bořek-Dohalská L, Frei E, Stiborová M (2004). Collect Czech Chem Commun.

[35] Poljaková J, Eckschlager T, Hrabeta J, Hrebacková J, Smutný S, Frei E, Martínek V, Kizek R, Stiborová M (2009). Biochem Pharmacol.

[36] Stiborova M, Poljakova J, Mrizova LI, Borek-Dohalska L, Eckschlager T, Adam V, Kizek R, Frei E (2014). Int J Electrochem Sci.

[37] Wohak LE, Krais AM, Kucab JE, Stertmann J, Øvrebø S, Seidel A, Phillips DH, Arlt VM (2014). Arch Toxicol.

[38] Hanna IH, Teiber JF, Kokones KL, Hollenberg PF (1998). Arch Biochem Biophys.

[39] Hanna IH, Reed JR, Guengerich FP, Hollenberg PF (2000). Arch Biochem Biophys.

[40] Culka M, Milichovsky J. Jerabek P, Stiborova M, Martinek V (2015) Neuro Endocrinol Lett 36 Suppl 1:29 26757119

[41] Shimada T, Wunsch RM, Hanna IH, Sutter TR, Guengerich FP, Gillam EMJ (1998). Arch Biochem Biophys.

[42] Guengerich FP, Martin MV (2006). Methods Mol Biol.

[43] Harnastai IN, Gilep AA, Usanov SA (2006). Protein Expr Purif.

[44] Lee SH, Yu HJ, Lee S, Ryu D-Y (2015). Toxicol Lett.

[45] Omura T, Sato R (1964). J Biol Chem.

[46] Wiechelman KJ, Braun RD, Fitzpatrick JD (1988). Anal Biochem.

[47] Stiborova M, Martinek V, Rydlova H, Hodek P, Frei E (2002). Cancer Res.

[48] Stiborová M, Bieler CA, Wiessler M, Frei E (2001). Biochem Pharmacol.

[49] Indra R, Moserova M, Kroftova N, Sulc M, Martinkova M, Adam V, Eckschlager T, Kizek R, Arlt VM, Stiborova M (2014). Neuro Endocrinol Lett.

[50] Moserová M, Kotrbová V, Aimová D, Sulc M, Frei E, Stiborová M (2009). Interdiscip Toxicol.

[51] Ječmen T, Ptáčková R, Černá V, Dračínská H, Hodek P, Stiborová M, Hudeček J, Šulc M (2015). Methods.

