# Supplementary material for: Heterologous expression of human cytochrome P450 2S1 in Escherichia coli and investigation of its role in metabolism of benzo[a]pyrene and ellipticine
Source: Monatsh Chem. 2016 Mar 30;147:881–8. doi: 10.1007/s00706-016-1738-2 (PMC4828499; doi:10.1007/s00706-016-1738-2)

**Supplementary Material**

Heterologous expression of human cytochrome P450 2S1 in *Escherichia coli* and investigation of its role in metabolism of benzo[a]pyrene and ellipticine

**Iveta Mrízová ● Michaela Moserová ● Jan Milichovský ● Miroslav Šulc ● René Kizek ● Kateřina Kubáčková ● Volker M. Arlt ● Marie Stiborová**

**Supplementary Table 1.** Mass spectrometry analysis of tryptic CYP2S1 peptides with verified identity/sequence by tandem mass spectrometry (MS/MS).

| **TRPM1-NT** | | ***m/z* signal** | |
| --- | --- | --- | --- |
| **Peptide Sequence [modification]** | **Numbering** | **Experimental** | **Calculated** |
| FTMLALR | 109-115 | 851.442 | 851.4813 |
| KFTMLALR | 108-115 | 979.526 | 979.5763 |
| DLVDAFLLK | 246-254 | 1033.553 | 1033.5934 |
| HPEEFNPDR | 387-395 | 1140.632 | 1140.5074 |
| LLALVPMGIPR | 342-352 | 1179.678 | 1179.7288 |
| LLALVPMoxGIPR | 342-352ox | 1195.686 | 1195.7237 |
| EGEELIQAEAR | 123-133 | 1244.551 | 1244.6123 |
| ELGAGQAPSLGDR | 314-326 | 1270.573 | 1270.6391 |
| EALGGQAEEFSGR | 68-80 | 1350.559 | 1350.6290 |
| LPYTDAVLHEAQR | 329-341 | 1512.710 | 1512.7811 |
| FSYEDKEFQAVVR | 170-182 | 1617.699 | 1617.7913 |
| QLLHHVSTLAAFTVR | 215-229 | 1692.876 | 1692.9549 |
| QVQQHQGNLDASGPAR | 230-245 | 1705.757 | 1705.8370 |
| TRLPYTDAVLHEAQR | 327-341 | 1769.885 | 1769.9299 |
| EELNRELGAGQAPSLGDR | 309-326 | 1911.865 | 1911.9524 |
| GKLPPGPTPLPLLGNLLQLR | 9-28 | 2094.178 | 2094.2804 |
| GTVAMLEGTFDGHGVFFSNGER | 81-102 | 2327.996 | 2328.0719 |
| QVQQHQGNLDASGPARDLVDAFLLK | 230-254 | 2720.315 | 2720.4120 |
| YGPVFTIYLGPWRPVVVLVGQEAVR | 43-67 | 2815.34 | 2815.5663 |
| LPPGPTPLPLLGNLLQLRPGALYSGLMR | 11-38 | 2954.544 | 2954.7018 |
| LPPGPTPLPLLGNLLQLRPGALYSGLMoxR | 11-38ox | 2970.574 | 2970.6967 |
| GKLPPGPTPLPLLGNLLQLRPGALYSGLMR | 9-38 | 3139.666 | 3139.8182 |
| GKLPPGPTPLPLLGNLLQLRPGALYSGLMoxR | 9-38ox | 3155.66 | 3155.8132 |
| AAGGTLLGVSSQGGQTYEMFSWFLRPLPGPHK | 183-214 | 3389.537 | 3389.7105 |
| GYTLPQGTEVFPLLGSILHDPNIFKHPEEFNPDR | 362-395 | 3877.673 | 3877.9553 |

Peptide sequence is shown in capital letter as a one letter amino acid code and ox – marks the oxidation of peptide.

**Supplementary Scheme 1.** The CYP2S1 protein sequence coverage determined by mass spectrometry analysis of tryptic peptides (determined value of 54%, 264 covered from total 488 amino acid residues).

**1 MAKKTSSKGK LPPGPTPLPL LGNLLQLRPG ALYSGLMRLS KKYGPVFTIY**

**51 LGPWRPVVVL VGQEAVREAL GGQAEEFSGR GTVAMLEGTF DGHGVFFSNG**

**101 ERWRQLRKFT MLALRDLGMG KREGEELIQA EARCLVETFQ GTEGRPFDPS**

**151 LLLAQATSNV VCSLLFGLRF SYEDKEFQAV VRAAGGTLLG VSSQGGQTYE**

**201 MFSWFLRPLP GPHKQLLHHV STLAAFTVRQ VQQHQGNLDA SGPARDLVDA**

**251 FLLKMAQEEQ NPGTEFTNKN MLMTVIYLLF AGTMTVSTTV GYTLLLLMKY**

**301 PHVQKWVREE LNRELGAGQA PSLGDRTRLP YTDAVLHEAQ RLLALVPMGI**

**351 PRTLMRTTRF RGYTLPQGTE VFPLLGSILH DPNIFKHPEE FNPDRFLDAD**

**401 GRFRKHEAFL PFSLGKRVCL GEGLAKAELF LFFTTILQAF SLESPCPPDT**

**451 LSLKPTVSGL FNIPPAFQLQ VRPTDLHSTT QTRHHHHH**

**Supplementary Figure 1.** HPLC analysis of BaP-7,8-dihydrodiol metabolites formed by human recombinant CYP2S1 in the presence of cumene hydroperoxide. BaP-7,8,9,10-tetrahydrotetrol was identified using an authentic standard (see Supplementary Fig. 2).


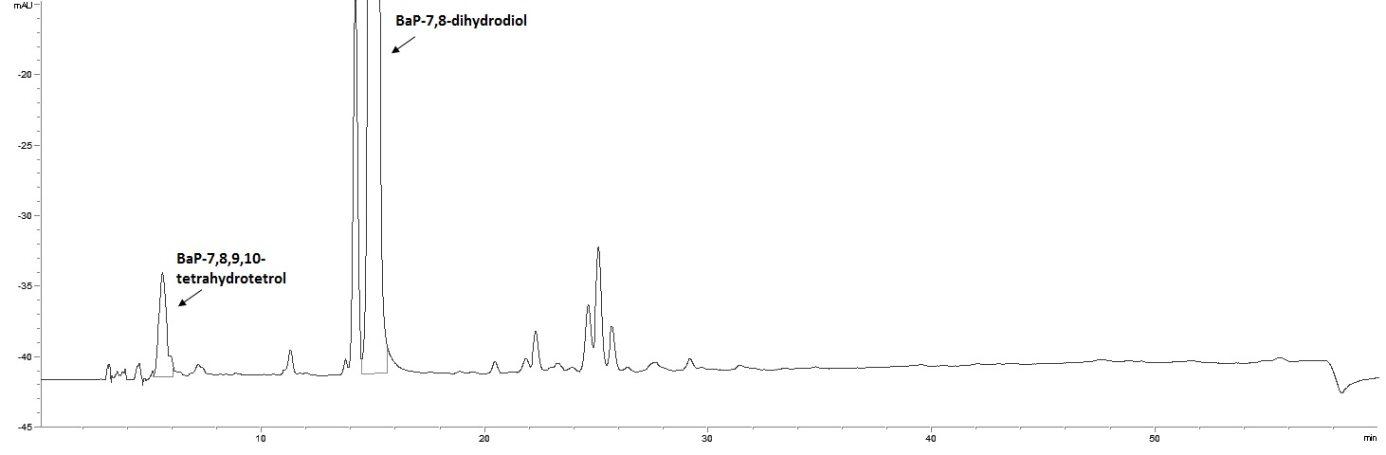


**Supplementary Figure 2.** Spectrum of BaP-7,8,9,10-tetrahydrotetrol formed by human CYP2S1. The spectrum was identical with that of authentic standard.


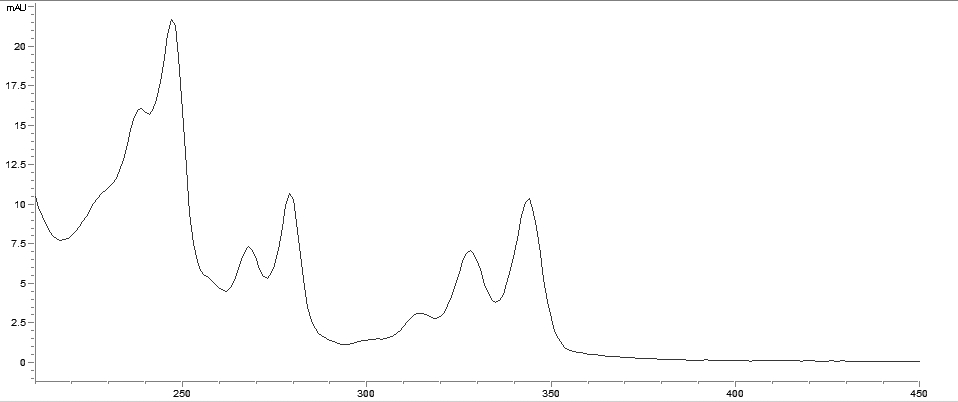

Supplement: Supplementary file 1 — Supplementary material 1 (DOCX 132 kb) [file 706_2016_1738_MOESM1_ESM.docx]
